# Supplementary material for: Genome-wide analyses identify 21 infertility loci and over 400 reproductive hormone loci across the allele frequency spectrum
Source: medRxiv. 2024 Mar 20:2024.03.19.24304530. Preprint. [Version 1] doi: 10.1101/2024.03.19.24304530 (PMC10984039; doi:10.1101/2024.03.19.24304530)
Supplement: Supplement 2 [file media-2.pdf]

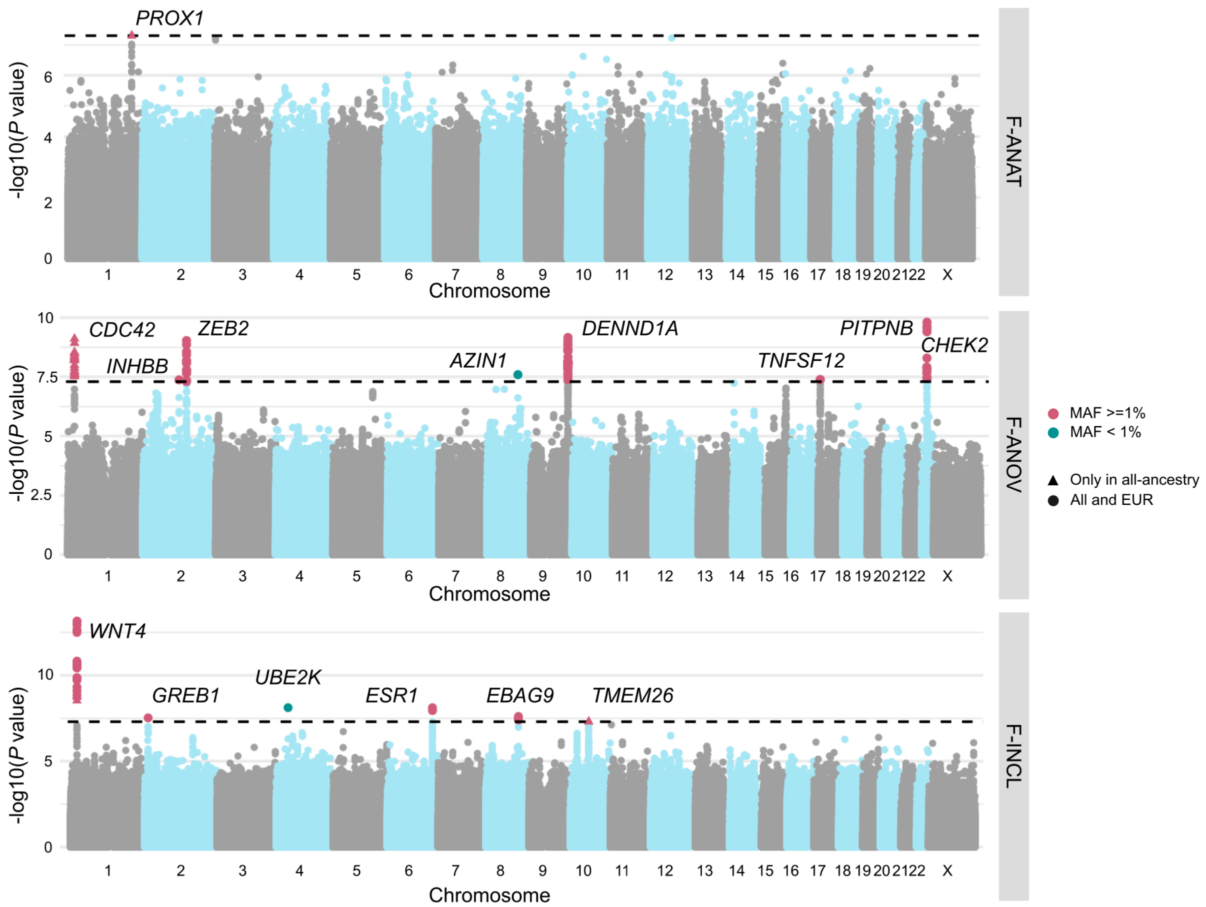

Supp. Figure 1. Manhattan plots for female infertility GWAS meta-analyses not presented in the main text. Genetic variants associated with anatomical female infertility (F-ANAT) (top), anovulatory female infertility (F-ANOV) (middle), and idiopathic infertility (unknown causes) defined by inclusion of a code for idiopathic infertility (F-INCL) (bottom). Each point depicts a single SNP, with genome-wide significant (GWS) SNPs (P < 5E-08, dashed line) coloured in pink for common variants with minor allele frequency (MAF)>=1% and green for those with MAF<1%. Triangles represent SNPs that only reach GWS in all-ancestry GWAS meta-analyses. SNPs are annotated with the mapped gene.

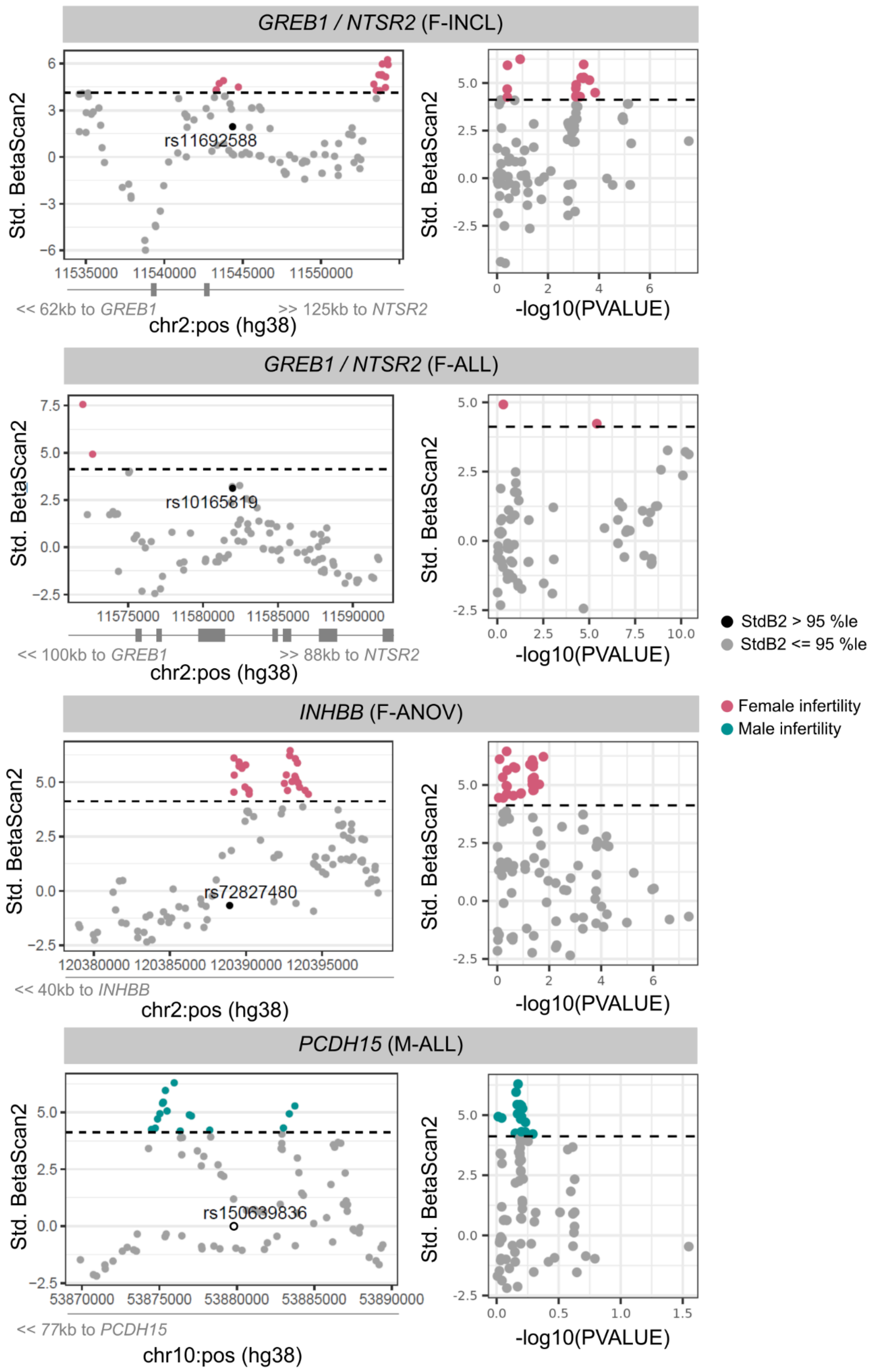

Supp. Figure 2. Balancing selection, as measured by standardised BetaScan2 (StdB2) scores, at infertility-associated loci. Each panel displays windows of +/- 10 kb around a lead infertility-associated variant, annotated with nearest gene and location: rs10165819 (F-ALL), rs72827480 (anovulatory infertility, F-ANOV), rs11692588 (female idiopathic infertility by inclusion, F-INCL), and rs150639836 (male infertility of all causes, M-ALL). Dashed lines indicate 95th %ile of

StdB2, and variants crossing this threshold are coloured in pink (for female infertility loci) or green (for the male infertility locus). Left: Locus plots depicting genomic position on the x-axis and StdB2 on the y-axis. The lead variant rs150639836 (open circle) is not present in the StdB2 dataset and thus assigned StdB2 of 0. Right: Scatter plots depicting relationship between -log10 of the GWAS p-value for the variant association with infertility on the x-axis and StdB2 on the y-axis.

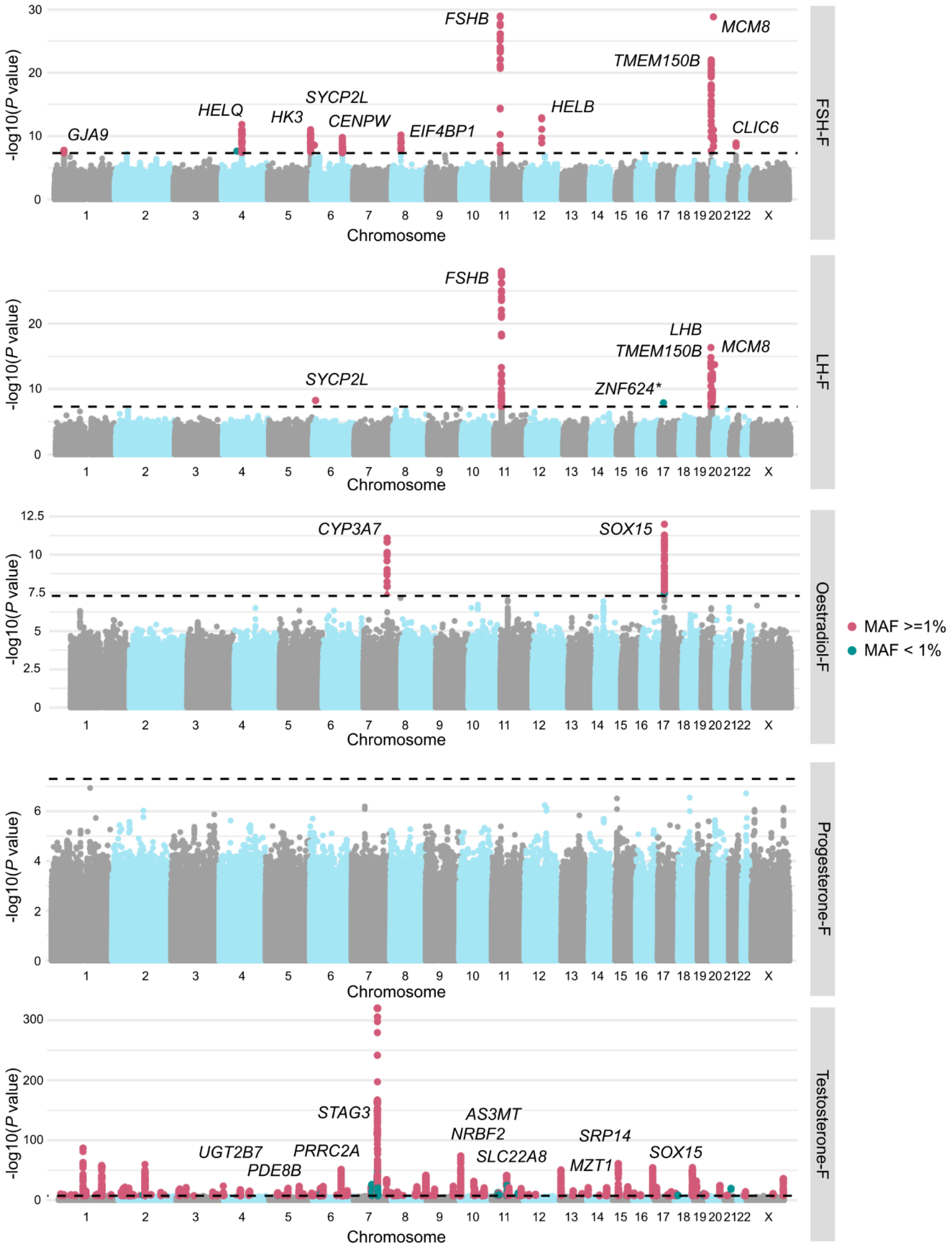

Supp. Figure 3. Manhattan plots for female reproductive hormone GWAS meta-analyses. Each panel displays genetic variants associated with a different reproductive hormone, from top to bottom: follicle stimulating hormone (FSH), luteinising hormone (LH), oestradiol, progesterone, and total testosterone. Each point depicts a single SNP, with genome-wide significant (GWS) SNPs (P < 5E-08, dashed line) coloured: in pink for common variants with minor allele frequency (MAF)>=1% and green for those with MAF<1%. SNPs are annotated with the mapped gene; for testosterone, only novel SNPs (ten lowest P-values) are annotated. * indicates that lead variant is present in only one study. For all lead variants, refer to Supp. Table 10.

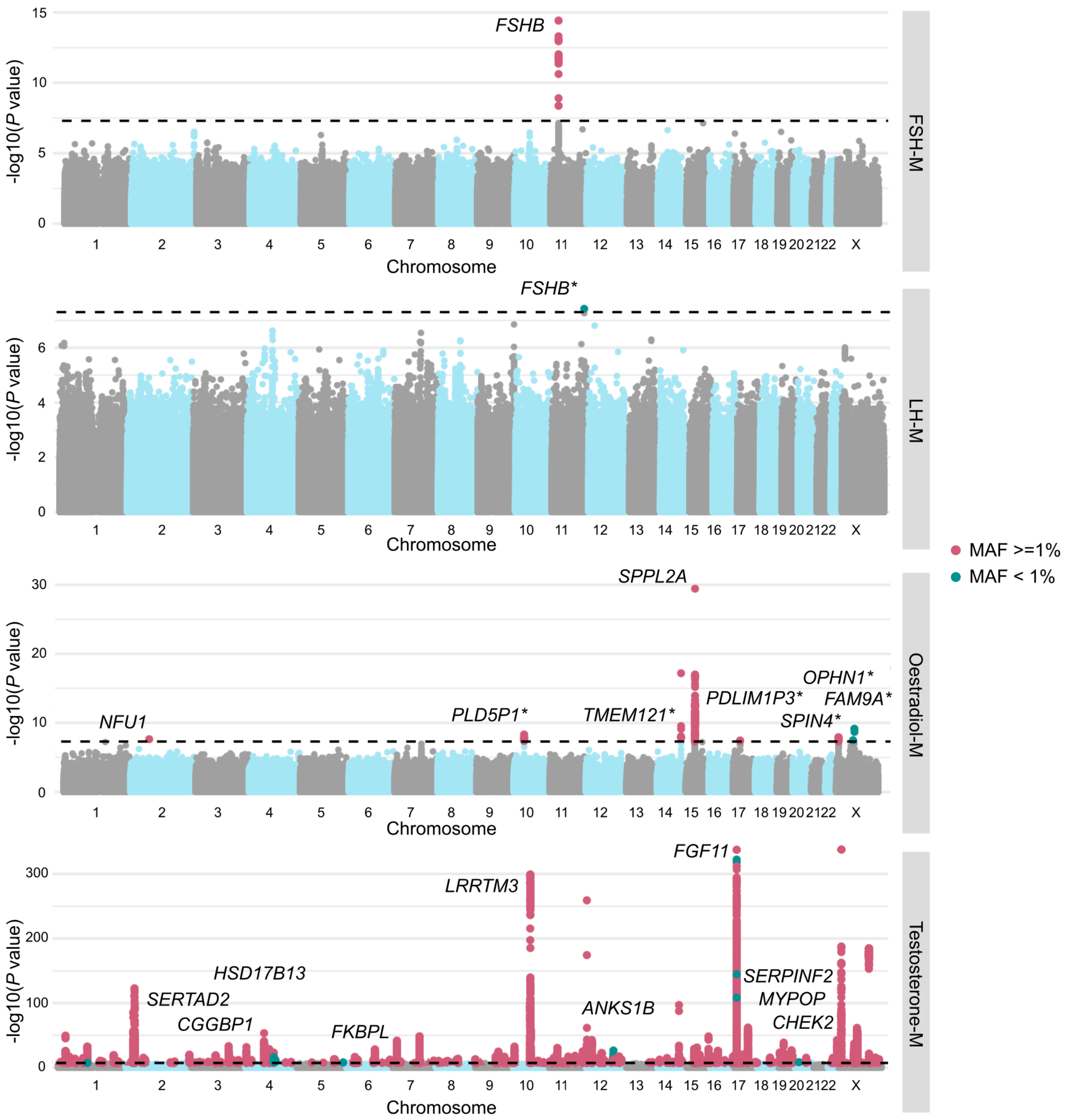

Supp. Figure 4. Manhattan plots for male reproductive hormone GWAS meta-analyses. Each panel displays genetic variants associated with a different reproductive hormone, from top to bottom: follicle stimulating hormone (FSH), luteinising hormone (LH), oestradiol, and total testosterone. Each point depicts a single SNP, with genome-wide significant (GWS) SNPs (P < 5E-08, dashed line) coloured: in pink for common variants with minor allele frequency (MAF)>=1% and green for those with MAF<1%. SNPs are annotated with the mapped gene; for testosterone, only novel SNPs (ten lowest P-values) are annotated. * indicates that lead variant is present in only one study. For all lead variants, refer to Supp. Table 10.

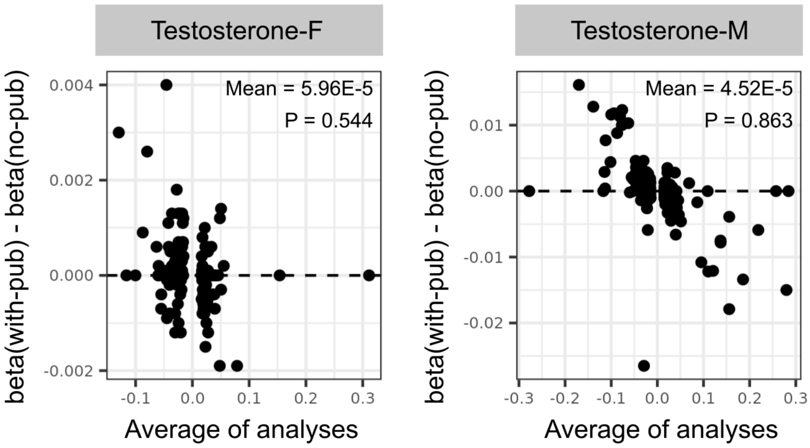

Supp. Figure 5. Comparison of effect sizes of SNPs associated with testosterone in main meta-analyses and sensitivity analyses without publicly available summary statistics. Variants that are genome-wide significant (GWS *P* < 5E-08) in the main GWAS with public data are plotted. The Bland-Altman plot displays the difference between effect sizes estimated in the with-public data (with-pub) and no-public data (no-pub) GWASs for each variant, plotted against the mean estimate from the two sets of analyses. The mean difference and one-sample t-test P-value (with null hypothesis of mean difference = 0) are displayed for each of the strata. Left: female-specific analyses, Right: male-specific analyses, from all-ancestry samples.

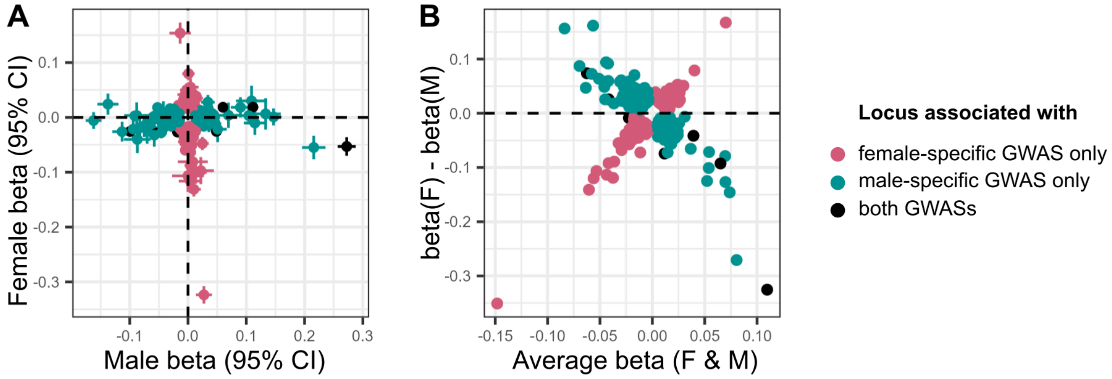

Supp. Figure 6. Sex heterogeneity in the effects of lead variants associated with testosterone. Lead variants in either female-specific testosterone GWAS (coloured in pink), male-specific testosterone GWAS (coloured in green), or both (coloured in black) are displayed. Results are from all-ancestry meta-analyses. (A) Scatter plot comparing effect sizes and 95% confidence intervals (CIs), plotted as error bars, of GWS variants. (B) Bland-Altman plot displaying the difference between effect sizes estimated in the female-specific and male-specific GWASs for each variant, plotted against the mean estimate from both GWASs.

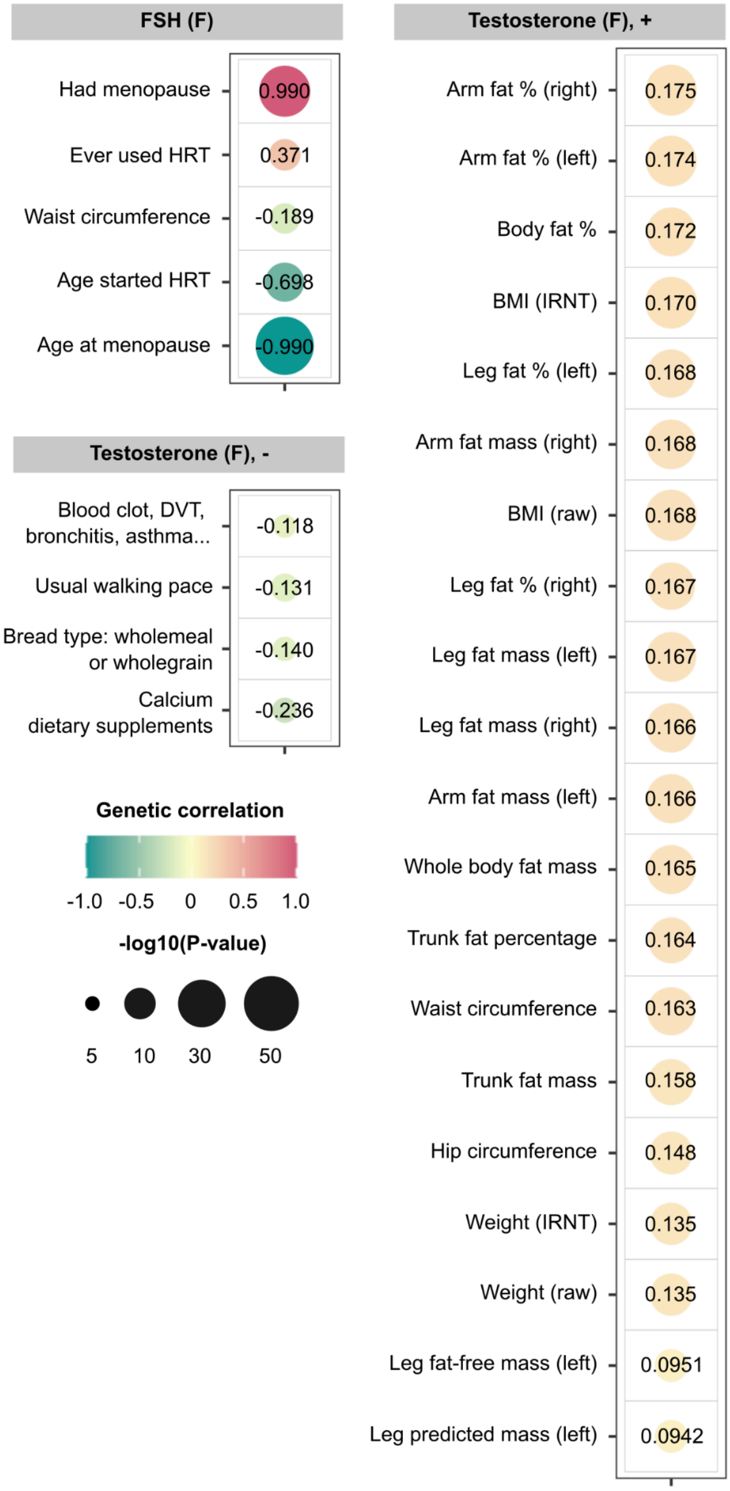

Supp. Figure 7. Genetic correlations (r_g) between follicle stimulating hormone in females (FSH-F) or testosterone-F and traits across the phenome. Trait summary statistics were generated by the Neale lab[53] and SNP-based genetic correlation calculations were performed using the LDSC software on a subset of 1 million HapMap3 SNPs[52]. Points are coloured by r_g estimate and sized by -log10(*P*). Only phenotypes associated at $P < 2.45\text{E-}05$ (FWER controlled at 5% across 2,040 tests using the Bonferroni method, accounting for 340 effectively independent UKBB phenotypes and 6 hormone or infertility strata are displayed, up to a maximum of 20 phenotypes. Phenotypes in the panel labelled "Testosterone (F), -" are negatively correlated with testosterone-F, and those in the panel labelled "Testosterone (F), +" are positively correlated with testosterone-F. HRT = hormone

replacement therapy, BMI = body mass index, IRNT = inverse-rank normally transformed, "Blood clot, DVT, bronchitis, asthma…" = Blood clot, deep vein thrombosis (DVT), bronchitis, emphysema, asthma, rhinitis, eczema, Hayfever, allergic rhinitis or eczema allergy diagnosed by doctor.

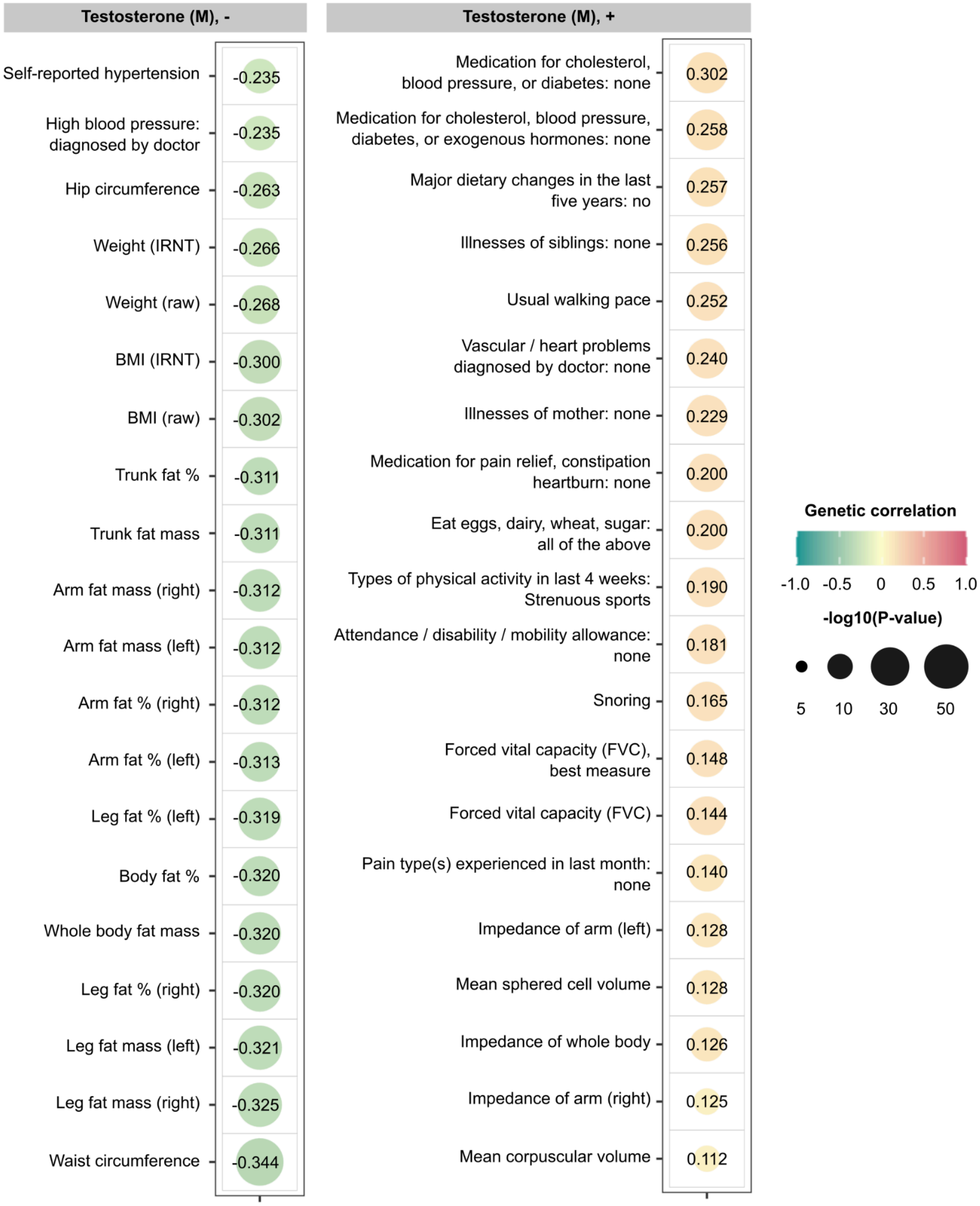

Supp. Figure 8. Genetic correlations (r_g) between testosterone in males and traits across the phenome. Trait summary statistics were generated by the Neale lab[53] and SNP-based genetic correlation calculations were performed using the LDSC software[51] on a subset of 1 million HapMap3 SNPs[52]. Points are coloured by r_g estimate and sized by -log10(P). Only phenotypes associated at P

< 2.45E-05 (FWER controlled at 5% across 2,040 tests using the Bonferroni method, accounting for 340 effectively independent UKBB phenotypes and 6 hormone or infertility strata are displayed, up to a maximum of 20 phenotypes. Phenotypes in the panel labelled “Testosterone (M), -” are negatively correlated with testosterone-M, and those in the panel labelled “Testosterone (M), +” are positively correlated with testosterone-M. BMI = body mass index, IRNT = inverse-rank normally transformed.

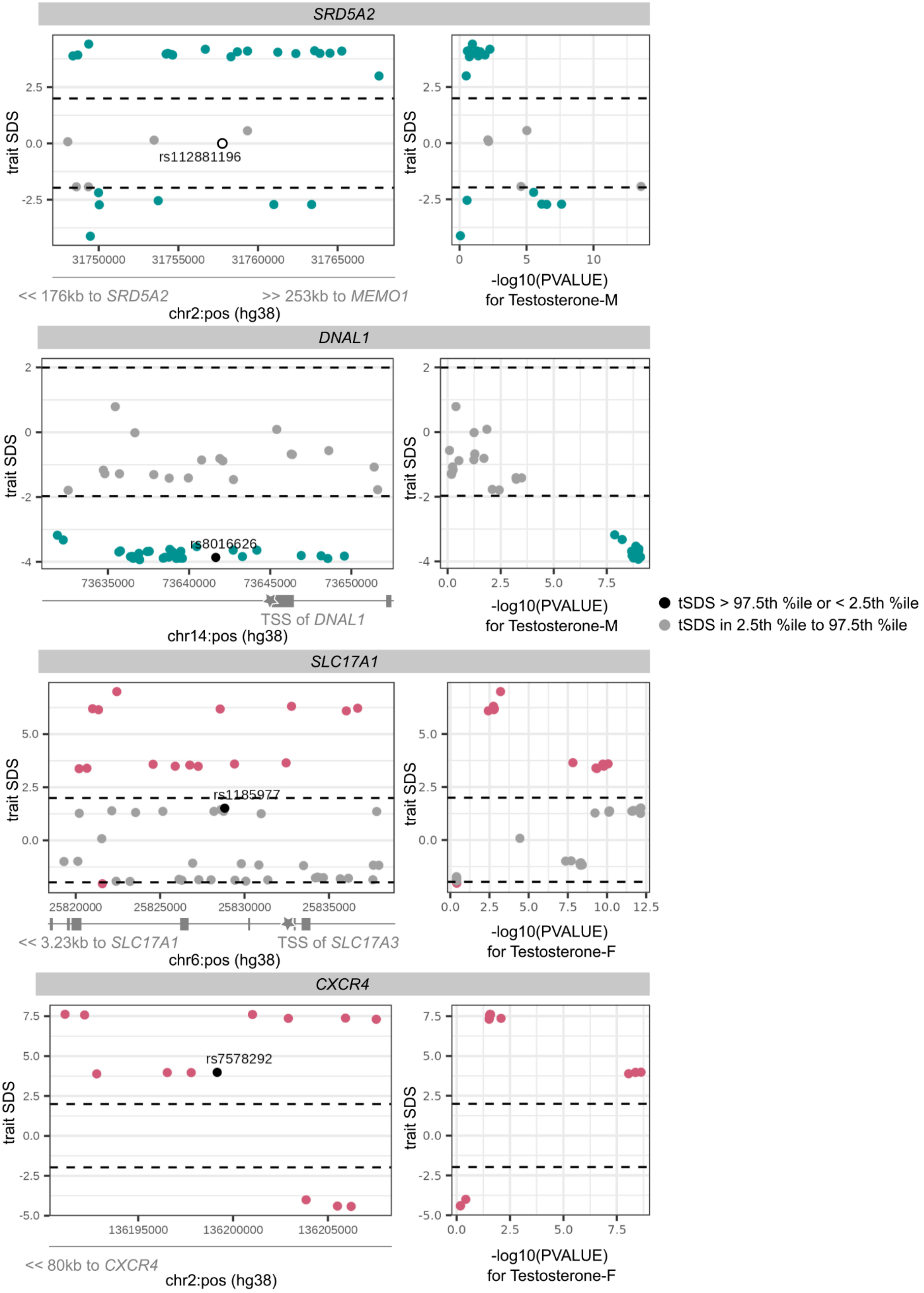

Supp. Figure 9. Trait-aligned Singleton Density Scores (SDSs), measuring recent directional selection, at testosterone-associated loci. Each panel displays windows of +/- 10 kb around a lead testosterone-associated variant, annotated with the location of nearest gene transcription start sites (TSSs) for all variants with extreme tSDSs: rs112881196 and rs8016626 (male-specific), and rs1185977 and rs7578292 (female-specific). The tSDSs are aligned to the testosterone-increasing

allele, wherein a positive tSDS indicates positive selection for testosterone-increasing allele at the locus. Dashed lines indicate 2.5th percentile (%ile) and 97.5th %ile of SDSs, and variants below or above this threshold respectively are coloured in green (for male-specific loci) and pink (for female-specific loci). Left: Locus plots depicting genomic position on the x-axis and trait-SDS on the y-axis. The lead variant rs112881196 (open circle) is not present in the tSDS dataset and thus assigned a score of 0. Right: Scatter plots depicting relationship between -log10 of the GWAS p-value for the variant association with testosterone on the x-axis and tSDS on the y-axis.

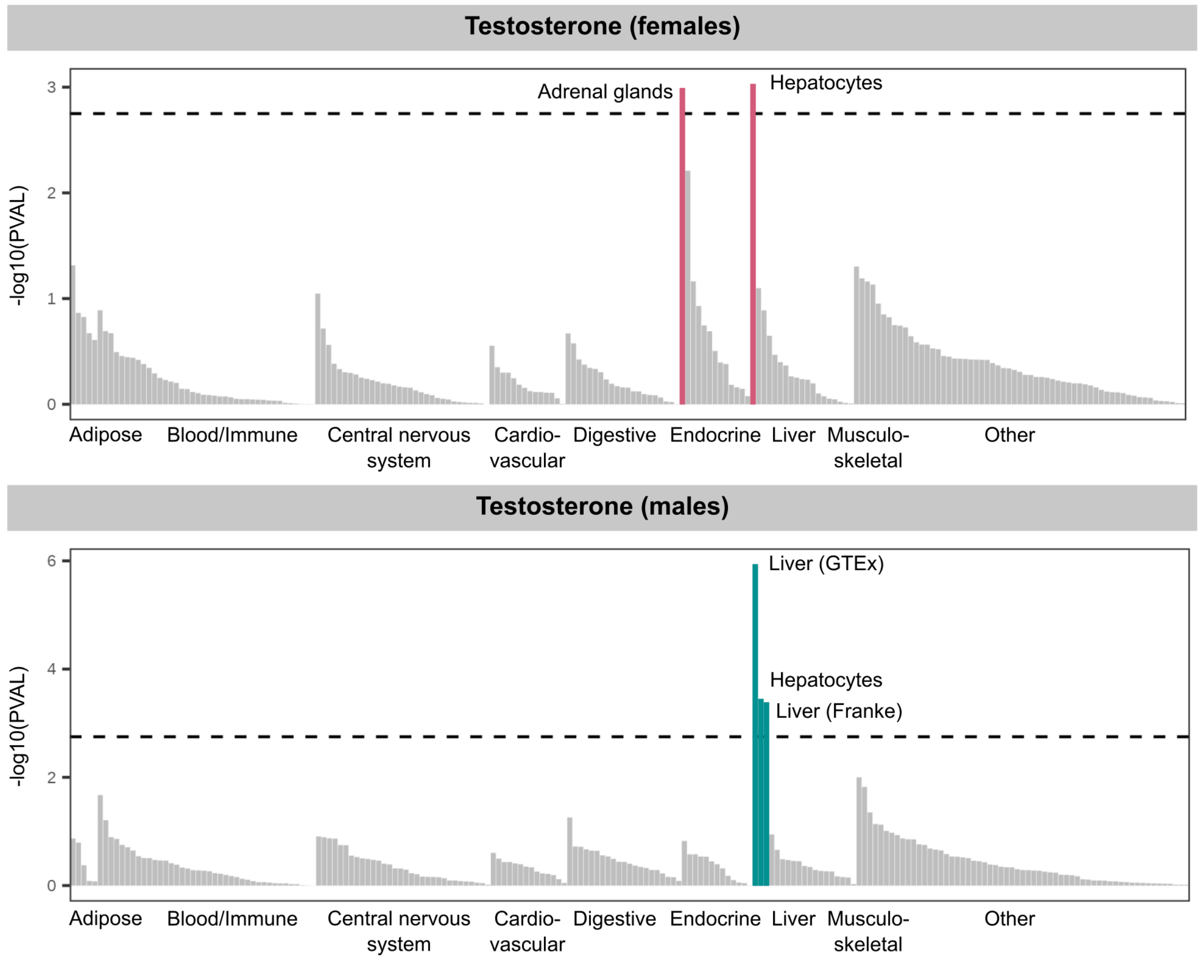

Supp. Figure 10. Enrichment of testosterone heritability across tissues and cell types. Partitioned heritability across 205 tissues and cell-types from the Genotype Tissue Expression (GTEx) Project database[41] and the Franke lab single-cell database[72] was assessed using partitioned LD-score regression[51]. Tissues and cell types are broadly grouped by organ system, and those that reach significance (FDR < 5%, dashed line), are annotated and coloured in: pink for testosterone in females (top) and green for testosterone in males (bottom).

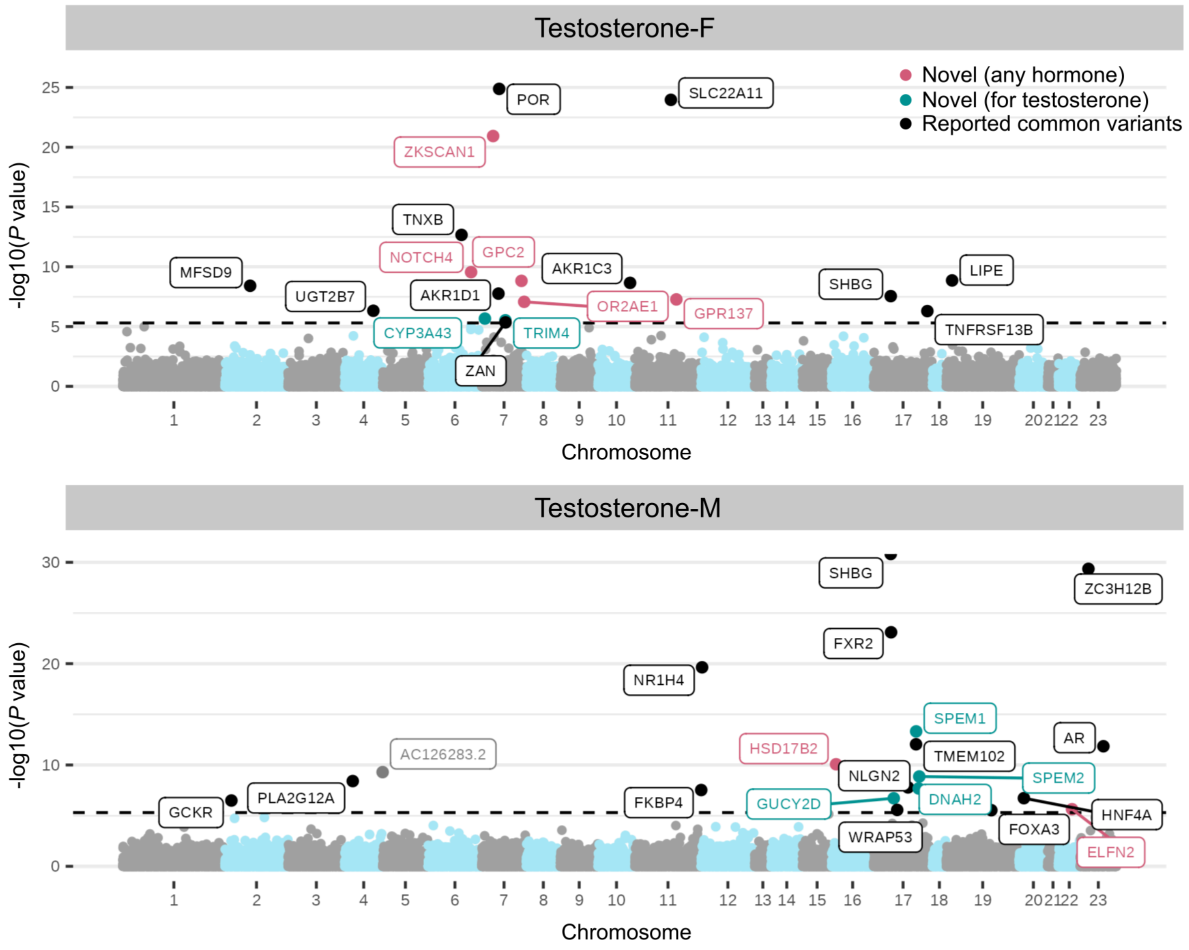

Supp. Figure 11. Gene-based Manhattan plots for burden of rare variants associated with testosterone in females and males in UK Biobank. Significance levels estimated using the Cauchy combined P-value are displayed, with significant genes (exome-wide significant at *P* < 5E-06 (FWER controlled at 5% using the Bonferroni method, across 10,000 effectively independent genes) coloured in: pink if no previous common variant associations with the gene have been reported for any of 28 reproductive hormones in the GWAS Catalog[62], green if no previous common variant associations with the gene have been reported for testosterone, and black otherwise.

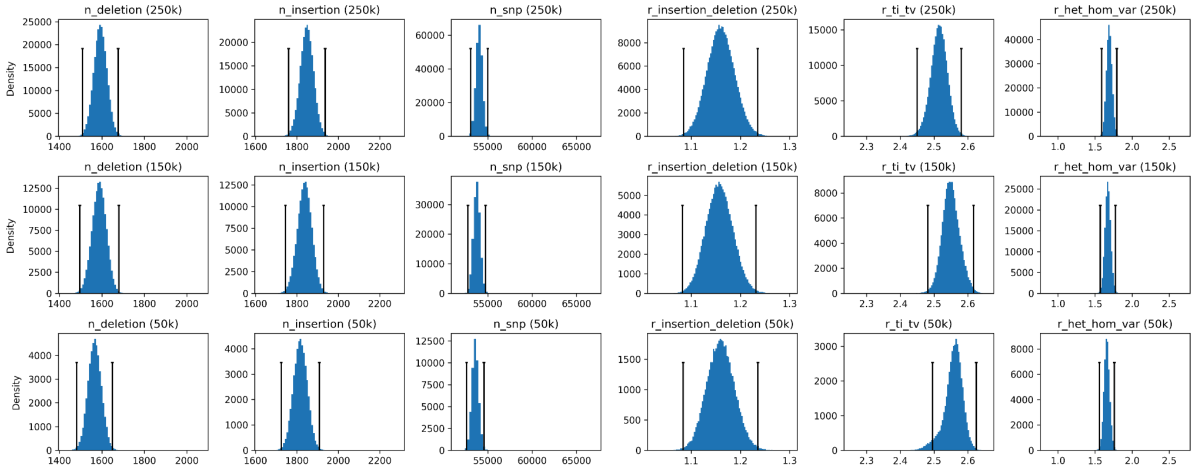

Supp. Figure 12. MAD thresholds for QC of exome sequencing samples. Samples with any of n_deletion, n_insertion, n_snp, r_insertion_deletion, r_ti_tv, and r_het_hom_var exceeding four MADs from the median are removed. MAD thresholds are displayed as vertical lines, conditional on tranche size (50k, 200k, 450k), from shortest to tallest.

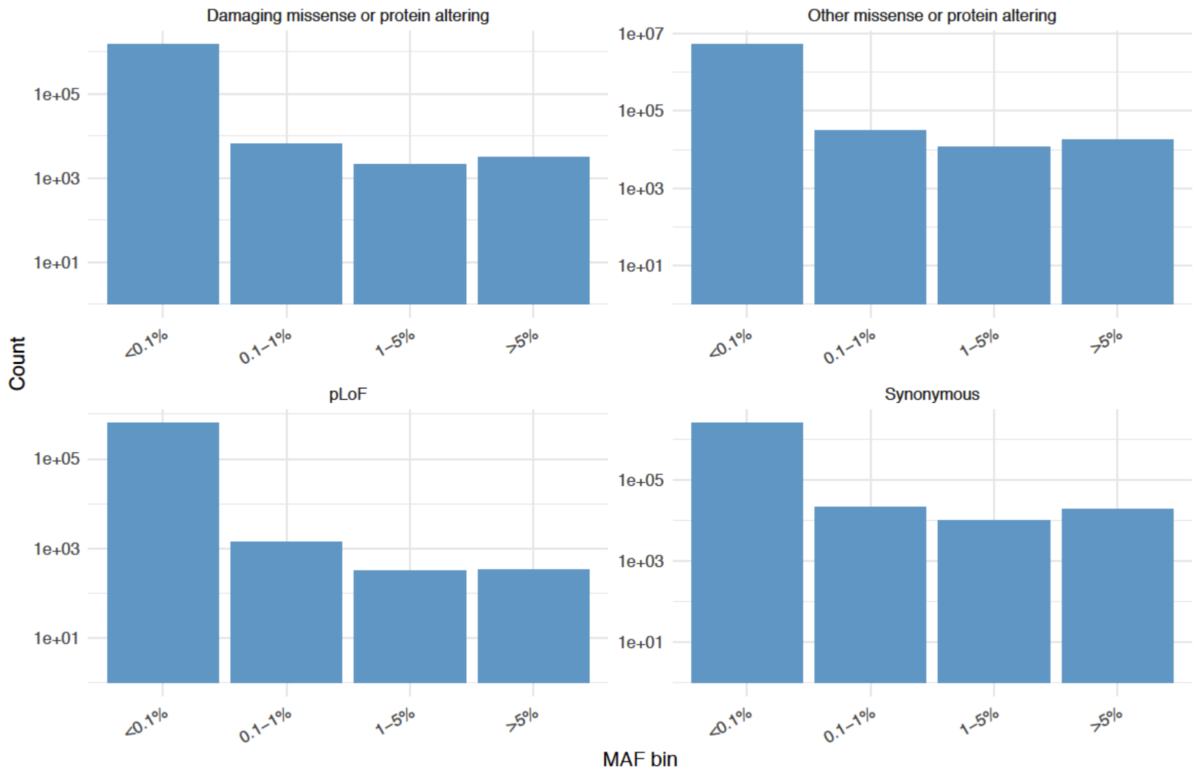

Supp. Figure 13. Average number of variants per individual, binned by MAF, according to the x-axes. Counts (y-axes) are on a log scale.

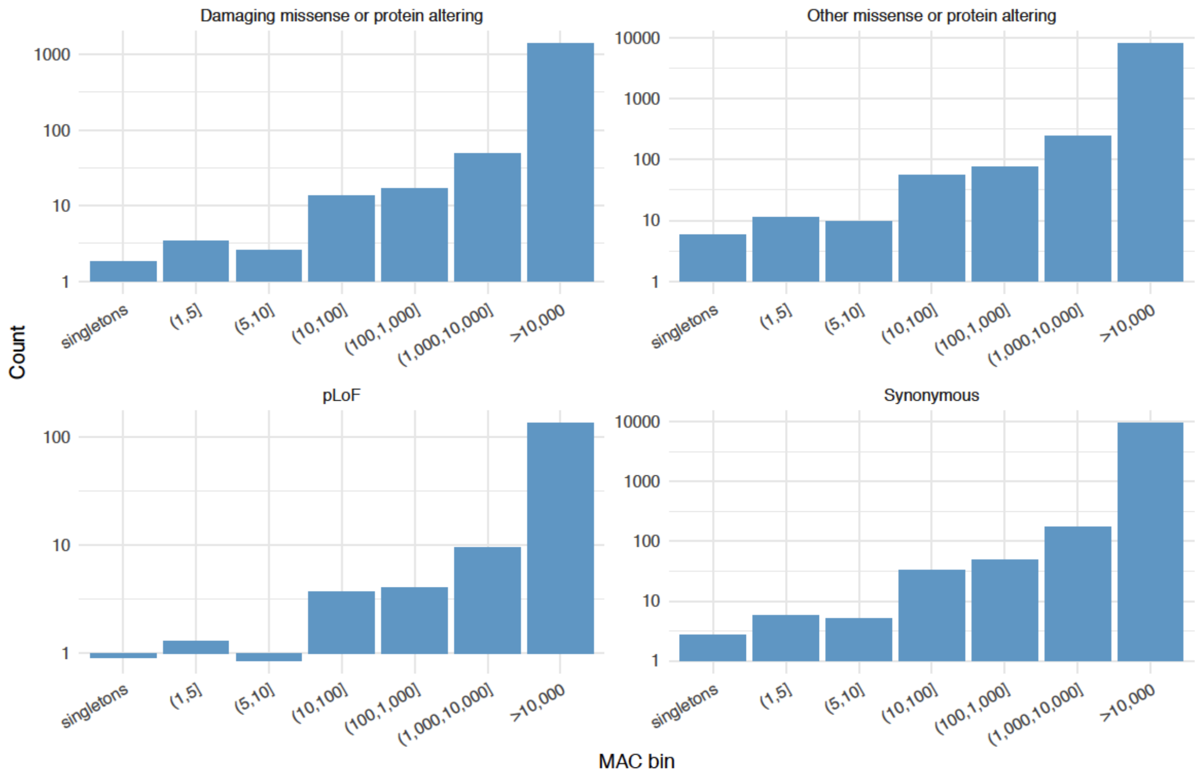

Supp. Figure 14. Average number of variants binned by minor allele count (MAC), per individual according to the *x*-axes. Count (y-axes) are on a log scale.
